# HPLC-UV-MS Profiles of Phenolic Compounds and Antioxidant Activity of Fruits from Three Citrus Species Consumed in Northern Chile

**DOI:** 10.3390/molecules191117400

**Published:** 2014-10-29

**Authors:** Anghel Brito, Javier E. Ramirez, Carlos Areche, Beatriz Sepúlveda, Mario J. Simirgiotis

**Affiliations:** 1 Laboratorio de Productos Naturales, Departamento de Química, Facultad de Ciencias Básicas, Universidad de Antofagasta, Av. Coloso S-N, Antofagasta 1240000, Chile; E-Mails: Anghel.Brito@gmail.com (A.B.); je.ramirez.007@gmail.com (J.E.R.); 2 Departamento de Química, Facultad de Ciencias, Universidad de Chile, Casilla 653, Santiago 7800024, Chile; E-Mail: areche@uchile.cl; 3 Departamento de Ciencias Químicas, Universidad Andrés Bello, Campus Viña del Mar, Quillota 980, Viña del Mar 2520000, Chile; E-Mail: bsepulveda@uc.cl

**Keywords:** endemic fruits, poliphenolics, quantitation, antioxidants, HPLC-MS, Pica lemon, Limón de Pica, chilean species

## Abstract

Peels and edible pulp from three species of citrus including *Citrus aurantifolia* (varieties pica and sutil) and *Citrus* x *lemon* var. Genova widely cultivated and consumed in Northern Chile (I and II region) were analyzed for phenolic compounds and antioxidant activity for the first time. A high performance electrospray ionization mass spectrometry (HPLC-UV-ESI-MS) method was developed for the rapid identification of phenolics in extracts from peels and juices of all species. Several flavonoids including one kaempferol-*O*-hexoside (peak **16**) and one hesperidin derivative (peak **22**) three quercetin derivatives (peaks **4**, **19** and **36**), five isorhamnetin derivatives (peaks **5**, **23**, **24**, **26** and **29**) four luteolin derivatives (peaks **14**, **25**, **27** and **40**), seven apigenin derivatives (peaks **2**, **3**, **12**, **20**, **34**, **35** and **39**), seven diosmetin derivatives (peaks **7**–**9**, **17**, **21**, **31** and **37**), three chrysoeriol derivatives (peaks **10**, **18** and **30**), and four eryodictiol derivatives (peaks **6**, **13**, **15** and **38**) were identified in negative and positive mode using full scan mass measurements and MS^n^ fragmentations. Ascorbic acid content was higher in the pulps of the varieties *Genova* and *Sutil* (60.13 ± 1.28 and 56.53 ± 1.06 mg ascorbic acid per g dry weight, respectively) while total phenolic content was higher in Pica peels followed by Sutil peels (34.59 ± 0.81 and 25.58 ± 1.02 mg/g GAE dry weight, respectively). The antioxidant capacity was also higher for Pica peels (10.34 ± 1.23 µg/mL in the DPPH assay and 120.63 ± 2.45 µM trolox equivalents/g dry weight in the FRAP assay). The antioxidant features together with the high polyphenolic contents can support at least in part, the usage of the peel extracts as nutraceutical supplements, especially to be used as anti-ageing products.

## 1. Introduction

**Figure 1:**
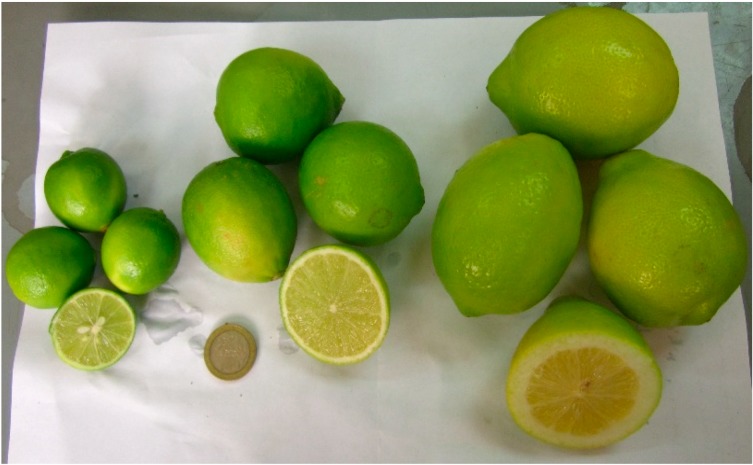
Pictures of Pica lemon (**right**), Sutil lemon (**middle**) and Genova lemon (**left**) from northern Chile.

The accumulation of oxidative damage due generally to intracellular ROS concentration has been proposed to be causal to ageing as defined by the Free radical Theory of Ageing, which has been subject to recent debate [1,2]. Fruits and vegetables are considered highly protective for human health against oxidative-stress related diseases and ageing [3]. There is evidence that a fruit extract with high vitamin C and polyphenol content was effective in decreasing intracellular ROS concentration and in protecting lipid, DNA and mitochondrial functionality from the damage induced by free radicals [4]. However, recent studies have demonstrated that the biological activities of polyphenols is not only related to their role as antioxidants but also to other pathways involved in cellular metabolism and cellular survival [5]. In this context and in the framework of the ageing process, citrus crops are one of the most important in the world, as producers of high vitamin C and polyphenolic content, and the fruits are among the most valuable functional diets, highly consumed globally in the form of fresh as well as processed juices and shown to lower oxidative stress-related diseases and ageing [6]. Furthermore, an extract of pummelo (*Citrus maxima*) was able to attenuate increased ROS levels in aged cells [7], while epidemiological studies reveal a strong correlation between high levels of citrus fruit consumption and low incidence of age-related diseases such as cardiovascular diseases and cancer [8]. Besides, citrus fruits showed cytoprotective action which is enhanced by the presence of antioxidants including vitamin C and flavonoids [9]. Citrus fruits are grown all over the world in more than 140 countries and production has increased enormously from an average of 48 million tons a year in the decade of 1970 to more than 100 million in 2005 [10]. Fruitpeels and seeds from lemon are major agro-industrial wastes in canned fruit manufacture and fruit juice. Peels are a source of molasses cold-pressed oils, flavonoids, pectin and also limonoids [11]. The flavonoids present in citrus pulps and peels are two main classes: the polymethoxylated flavones and the glycosylated flavanones [11,12,13,14]. These secondary metabolites exist in the form of flavanone glycosides mainly as diosmin, naringin, hesperidin and neohesperidin derivatives and possess anticonvulsion, antimutagenic, antiinflammatory, antioxidant, blood lipid lowering and anti-carcinogenic activities among others [8,12,15,16]. In Northern Chile (I and II region), the three cultivated and more widely consumed Citrus species are *limón de Pica* (Pica Lemon, *Citrus aurantifolia* (Christm) Swingle var Pica), a tart and tangy lime most famous for its use in Pisco Sour cocktails and produced mostly exclusively in the Town of Pica, which is located in an oasis in the Atacama desert, *limón Sutil* (*Citrus aurantifolia* (Christm) Swingle) var sutil and *limón Genova* (*Citrus* x *limon* (L.) Burm) var. Genova (Figure 1). In the last few years, several mixtures of phenolics have been analyzed with advanced LC-MS/MS equipment [17,18,19,20,21] including various publications dealing with the on-line HPLC-MS detection of flavonoids, phenolic acids and alkaloids in citrus fruits [12,13,14,15,22,23]. The advantage of HPLC-MS is its sensitivity and selectivity, and the ability to use tandem mass spectrometry for the observation of aglycones and diagnostic fragments [17,18]. As a continuation of our studies on antioxidant activity and phenolic composition of Chilean fruits [24,25], in the present work we have analyzed for the first time peels and edible pulps from three citrus varieties (pica, sutil and genova), highly consumed in northern Chile, for phenolic compounds using HPLC-UV-MS and compared their vitamin C content and antioxidant capacities. We demonstrate in this work that extracts from those citrus fruits have high polyphenolic content and antioxidant capacities and therefore could be useful as nutraceuticals or antioxidant supplements against oxidative stress and ageing.

## 2. Results and Discussion

### 2.1. Total Phenolic, Ascorbic Acid, Flavonoid Contents and Antioxidant Capacity of Citrus Extracts

**Figure 2:**
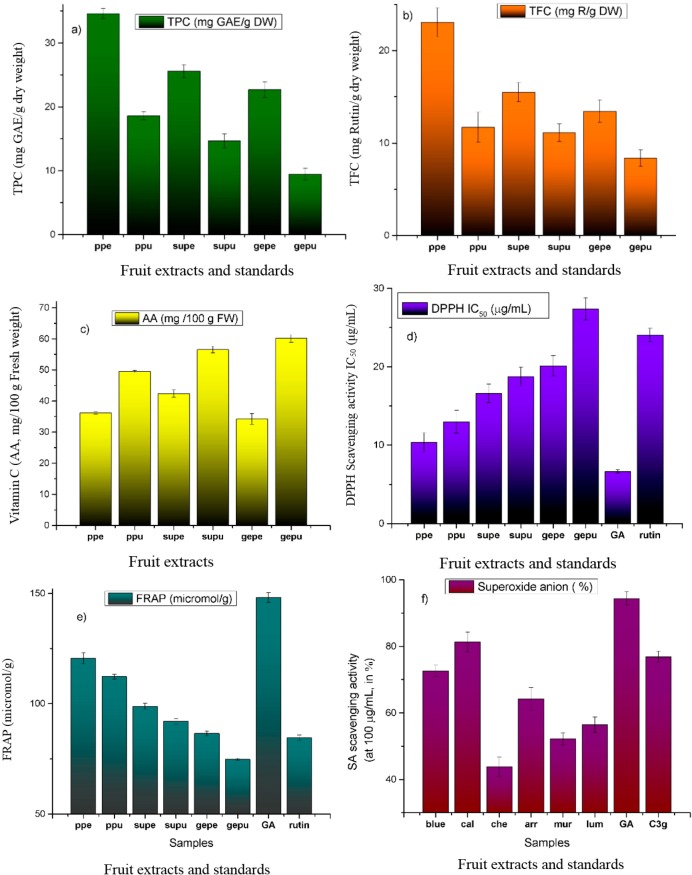
(**a**) Total Phenolic Content (TPC); (**b**) Total Flavonoid Content (TFC); (**c**) Ascorbic acid (AA) content; (**d**) Scavenging of the 1,1-diphenyl-2-picrylhydrazyl Radical (DPPH); (**e**) Ferric Reducing Antioxidant Power (FRAP) and (**f**) Superoxide Anion scavenging activity (SA), of peels and pulp of three citrus edible species from Chile.

In the present study, we assessed the polyphenolic profile of peels and pulps from citrus consumed in Chile, and evaluated their antioxidant capacity as well as the total phenolic, total flavonoid and vitamin C content by spectrophotometric methods. The fruits were collected from a local market in Antofagasta and the peels, pulp and seed-free juices were collected. The peels and pulps were extracted with methanol and the resulting extracts were processed by solid phase extraction. Antioxidant capacity of the extracts was measured and correlated with the total phenolic, flavonoid and ascorbic acid contents. Total flavonoid and phenolic contents were previously determined for several citrus [26] including non edible peels [10,27]. The methods for assessment of antioxidant effects were critically reviewed and the most important advantages and shortcomings of each method were highlighted because the capacity of antioxidants has been assessed by various methods, but they often give inconsistent and conflicting results since comparisons between laboratories are difficult [28,29]. Furthermore, several studies show that total phenolic compounds, ascorbic acid and antioxidant capacity strongly differed between genotypes of each individual fruit [30]. In this study antioxidant activity was determined by three *in vitro* commonly used assays, namely DPPH free radical scavenging assay, ferric reducing assay and superoxide anion scavenging activity (Figure 2), while ascorbic acid content and total phenolic content of fresh fruit juices were determined by volumetric and Folin-Ciocalteu reagent method respectively. We have employed these three different antioxidant assays plus the Folin-Ciocalteu method which is an electron transfer based assay and gives reducing capacity, for the quantification of the antioxidant capacities of the fruit extracts, since no single assay will accurately reflect all of the radical sources or all antioxidants in a mixed or complex plant extract. The total phenolic, vitamin C and flavonoid contents as well as the extraction yield and antioxidant capacity measured by the bleaching of DPPH radical and ferric reducing antioxidant power are given in Figure 2 and Table S1, (supplementary materials). Ascorbic acid was higher in *C. limon var Genova* while total phenolic content was higher in Pica peels (60.13 ± 1.28 mg/g ascorbic acid and 34.59 ± 0.81 mg/g GAE respectively, Figure 2). In the DPPH assay, the peels and pulp of Pica lemon exhibited stronger scavenging potential when compared to the other citrus varieties (Figure 2 and Table S1). TPC, TFC values and DPPH activity showed acceptable correlation (*R^2^* = 0.624 and 0.587, respectively, Figures S1 and S2) as well as good correlation was observed between antioxidant assays (Figures S3–S5). Ferric reducing potential was also higher in pica peels and pulp, followed by Sutil peel and pulp. The total flavonoid content of Sutil lemon peel was close to that reported for a sample of Citrus limon (L.) Burm., from Taiwan (11.9 ± 0.66 mg/g) [27] while the total phenolic content of Genova pulp was similar to that reported for an alcoholic extract of peels from Citrus limon (L.) Burm., from Algeria (12.11 ± 0.62 mg GAE/g dry weight) [10]. The vitamin C content of the pica peel and pulp were higher than that reported for Tahiti lime (6.84 ± 0.3 and 41.4 ± 0.9 mg AA/100 g fresh weight, respectively) and sweet lime (22.6 ± 0.8 and 60.2 ± 2.2 mg AA/100 g fresh weight, respectively) [31]. However, in this assay both peel and pulp of Chilean varieties Genova and Sutil showed higher AA content than pica lemon (Table 1). Genova pulp showed values three times higher to that reported for citrus lemon from China (233.44 ± 2.52 mg AA per liter) [32]. Several articles have shown that harvest times and environmental parameters (light conditions, temperature, irrigation, fertilization or cultivation systems) can affect the antioxidant capacity in fruits [30,33], thus further studies using several different parameters and on the cultivation of Citrus in Northern Chile should be carried out to show the variation on the production and accumulation of bioactive compounds and antioxidant activities of these fruits.

### 2.2. Identification of Phenolic Constituents in Citrus Fruits by HPLC-UV and ToF-ESI-MS/MS

**Figure 3:**
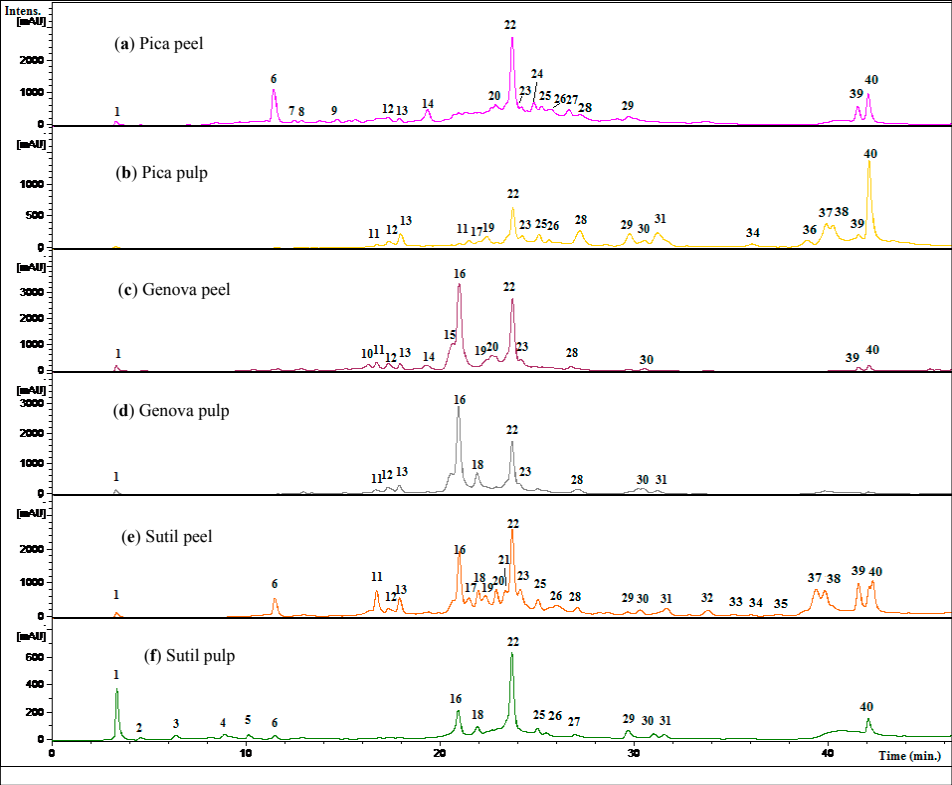
HPLC-UV chromatograms of Citrus fruits from northern Chile. (**a**) Pica lemon peel; (**b**) Pica lemon pulp (**c**) Genova lemon peel (**d**) Genova lemon pulp; (**e**) Sutil lemon peel and (**f**) Sutil lemon pulp, monitored at 280 nm. Peak numbers refer to those indicated in Table 1.

Phenolics occurring in pulp and peel extracts from three *Citrus* species consumed in northern Chile were separated by HPLC and UV/vis spectra were obtained using a diode-array detectorand electrospray mass spectrometry (ESI-MS) in full scan mode and tandem MS^n^ fragmentations. HPLC fingerprints were generated (Figure 3 and Figures S6–S8) and phenolic compounds subsequently analyzed by ESI-MS^n^ (Table 1).

Abreviations: ppe: pica peel, ppu: pica pulp, supe: sutil peel, supu: sutil pulp, gepe: genova peel; gepu: genova pulp, GA: gallic acid. Total phenolic content (a) expressed as mg gallic acid equivalents (GAE)/g dry weight; Total flavonoid content (b) expressed as mg quercetin/g dry weight; Ascorbic acid content (AA) (c) expressed as mg vitamin C/g dry weight; Antiradical DPPH activities (d) are expressed as IC_50_ in µg/mL for extracts and compounds; Ferric reducing power (FRAP) (e) expressed as µM trolox equivalents/g dry weight; Superoxide anion scavenging activity (SA) (f) expressed in percentage scavenging of SA at 100 µg/mL.

**Table 1:** Identification of phenolic compounds in *Citrus* fruits and aerial parts by LC-UV, LC-MS and MS/MS data.

Peak	Rt (min)	HPLC DAD λ Max (nm)	[M−H]^−^/[M+H]^+^ (*m/z*)	MS^2^ Ions (*m/z*)	MS^n^ Ions (*m/z*)	[2M−H]^−^ or [2M+H]^+^ (*m/z*)	Tentative Identification	Species/Plant Part
1	3.5	-	191	145	129, 111		Citric acid	Ppe, gpe, gpu, spe, spu
2	4.2	268, 334	739	431 (M-neohesperidose)	311 (431–120)		Apigenin 7-*O*-neohesperidoside-6-*C*-glucoside	Spu
3	17.5	268, 334	577/579	431	269, 225		Apigenin 7-*O*-neohesperidoside (Rhoifolin)	Supu
4	9.0	255, 354	771/773	609 (rutin)	463, 301, 179, 151		Quercetin 7-*O*-glucoside-3-*O*-rutinoside	Supu
5	10.1	250, 268, 342	639	315 (M-di-glucose)	301, 179, 151		Isorhamnetin-3-*O*-di-glucoside	Supu
6	10.9	285, 325	757	595	595, 449, 287		Eriodictyol-4'-*O*-neohesperidoside-7-*O*-glucoside	Spu, spe, ppe
7	12.0	250, 268, 342	623/625	503(M-120)	383, 312	1247	Diosmetin 6,8-di-*C*-glucoside (Lucenin-2,4'-methyl ether)	Ppe
8	12.2	250, 268, 342	607/609	563	299 (Diosmetin), 284		Diosmetin 7-*O*-neohesperidoside (Neodiosmin)	Ppe
9	14.1	250, 268, 342	607/609	563	299 (Diosmetin), 284		Diosmetin 7-*O*-neohesperidoside	Ppe
10	16.3		623/625	503(M-120)	383, 312	1247	Chrysoeriol 6,8-di-*C*-glucoside (Stellarin-2)	Gpe
11	16.2		357/359	194	151		3-(2-hydroxy-4-methoxyphenyl)-propanoic acid hexose	Ppu, gpe, gpu, spe, ppe
12	18.5	268, 334	577/579	431	269, 225, 201		Apigenin 7-*O*-rutinoside (Isorhoifolin)	Ppe, ppu, gpe, gpu, spe
13	31.5	285, 325	595	449 (M-rhamnose)	287 (M-neohesperidose)		Eriodictyol-7-*O*-neohesperidoside	Spu, spe, gpu, ppu
14	19.4	254, 267	593/595	285/287 (Luteolin)	241, 175	1187/1189	Luteolin 7-*O*-rutinoside	Ppe, gpe
15	19.1	285, 325	595/597	287/289 (eriodictyol)	151, 135, 107	1189/1191	Eriocitrin (Eriodictyol-7-*O*-rutinose)	Ppe, ppu, gpe, gpu, spe
16	23.5	250, 268, 342	607/609	563	299 (Diosmetin), 284		Diosmetin-7-*O*-rutinoside (Diosmin)	Gpe, gpu, spe, spu
17	21.0	250, 268, 342	461/463	341/343(M-120)	298		Diosmetin 8-*C*-glucoside (Orientin 4'-methyl ether)	Spe, ppu
18	22.0	255, 268, 345	461/463	341/343 (M-120)	298		Chrysoeriol 8-*C*-glucoside (Scoparin)	Spe, spu
19	22.3	254, 354	609/611	301 (M-rutinose)	179, 151		Rutin	Ppu, gpe, spe
20	23.0	268, 334	593/595	512	473 (M-120), 353 (M-240), 297		Apigenin 6,8-di-*C*-glucoside (Vicenin-2)	Ppe, gpe, spe
21	20.9	262, 362	593/595	285/287 (Kaempferol)		1187/1189	Kaempferol-3-*O*-Rutinose	spe
22	23.9	285, 332	609/700	301/303 (Hesperetin)	286, 177, 151		Hesperidin (hesperetin 7-*O*-rutinoside)	Ppe, ppu, gpe, gpu, spe, spu
23	24.1	250, 268, 342	623/625	315 (M-rutinose)	301, 179, 151		Isorhamnetin-3-*O*-rutinoside	Ppe, ppu, gpe, gpu, spe
24	24.7	250, 342	477	315 (M-glucose)	300, 179, 151		Isorhamnetin-3-*O*-glucoside	Ppe
25	25.0	254, 267	609/611	489 (M-120)	369 (M-240)		Luteolin 6,8-di-*C*-glucoside (Lucenin-2)	Ppe, ppu, spe, spu
26	26.0	250, 268, 342	461	301 (M-rhamnose)	301 (M-rhamnose)		Isorhamnetin-3-*O*-rhamnoside	Ppu, spe
27	27.1	254, 267	447	327 (M-120)	299		Luteolin 8-*C*-Glucoside (orientin)	Ppe, spu
28		285, 362	577/579	271	177, 151, 119, 107		Naringin (naringenin 7-*O*-neohesperidoside)	ppe, spe, gpu, ppu, gpe
29	29.7	250, 268, 342	491	315, 301	301		7-*O*-Methyl-Isorhamnetin-3-*O*-glucoside	Spu, spe, gpu, ppu
30	31.1	255, 268, 345	607/609	563	299 (Chrysoeryol), 284		Chrysoeriol 7-*O*-neohesperidoside	Spu, spe, gpu, ppu
31	20.0	250, 268, 342	461/463	341 (M-120)/343	298		Diosmetin-6-*C*-glucoside	Gpu, spe, gpe
32	33.8	254, 354	463	301 (M-glucose)	268, 179, 151		Quercetin 3-*O*-glucoside (Isoquercitrin)	Spe, ppu
33	27.2	268, 334	473/475	429	323, 161, 221		Apigenin 7-*O*-6''Acetyl-glucoside	Ppu, gpe, gpu, spe
34	38.9	268, 334	431/433	311 (M-120)	283		Apigenin-8-*C*-glucoside (vitexin)	Spe
35	40.1	268, 334	431/433	311 (M-120), 283	283		Apigenin-6-*C*-glucoside (isovitexin)	ppu
36	38.8	254, 354	301	179, 151	179, 151		Quercetin	Ppu, spe
37		250, 268, 342	299	179, 151	179, 151		Diosmetin	ppu
38	41.8	285, 325	287	179	151, 135, 125, 107		Eriodictyol	Gpe, ppu, spe
39	42.0	268, 334	269	179	225, 201, 151		Apigenin	gpe, spe, ppe
40	42.0	254, 267	285/287	269 (M-16)	243, 241, 217		Luteolin	Ppe, ppu, gpe, spe, spu, spe

Species and fruit parts: ppe: pica peel; ppu: pica pulp; gpe: genova peel; gpu: genova pulp; spe: sutil peel; spu: sutil pulp.

**Figure 4:**
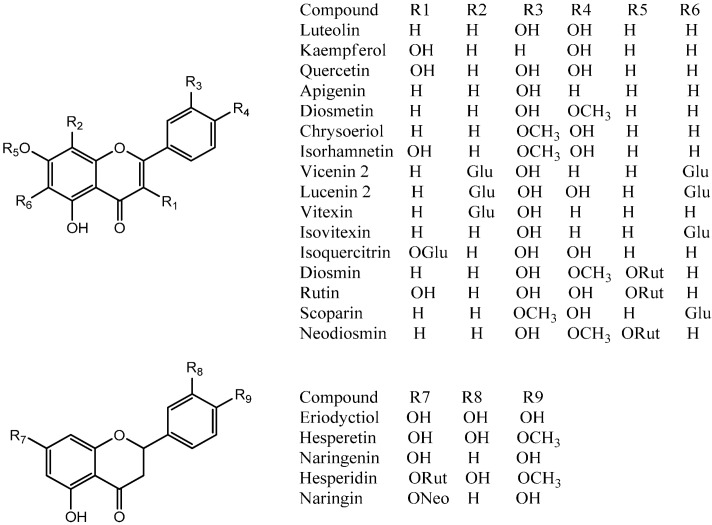
Structures of main flavonoids identified in Citrus species from Northern Chile.

**Figure 5:**
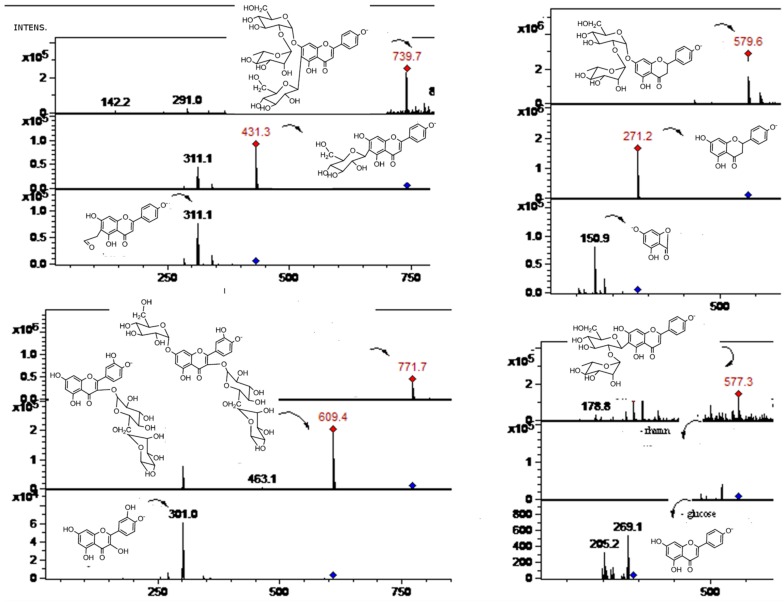
MS spectra of peaks **2**, **4**, **12**, and **28**. (Refer to Table 1).

**Figure 6:**
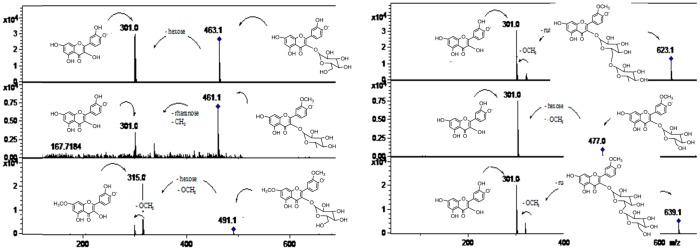
MS spectra of peaks **5**, **23**, **24**, **26**, **29** and **32**. (Refer to Table 1).

Preliminary analysis of UV-vis spectra obtained for the peaks gave a first indication of the family of phenolic compounds [34,35,36,37]. The structures of these compounds were proposed based on UV maxima (Table 1) as well as fragmentation pattern using ESI-MS-MS experiments (key aglycone fragments of 179 and 151 for quercetin and its methyl derivatives, 257, 243 for kaempferol, 241, 175 for luteolin, 225, 201, 149 for apigenin, 151, 135 for eriodictyol and 151, 177 for naringenin) [38]. Some compounds were identified by co-elution with standards while several flavonoids such as diosmin, diosmetin, naringin, vitexin, hesperidin and their MS^n^ fragments (Table 1) were coincident with those compounds already reported to occur in several Citrus species [12,13,14,15,22,23]. In addition, the loss of 162 Daltons was indicative of hexose (glucose or galactose, the most common sugars found in flavonoids) the loss of 146 Daltons was indicative of rhamnose, the loss of 133 Daltons was indicative of pentose (xylose or arabinose, the most common pentoses found in natural products), and the loss of 308 Daltons indicative of compounds having the disaccharide structure rutinose or neohesperidose linked thorough an -*O*-glycosidic bond [39]. In the MS identification of *C*-glycosides, the key fragmentations used were [M-60]^−^, [M-90]^−^, [M-120]^−^ and [M-240]^−^ [13], while branched *O*- and *C*-glucosides were identified as described previously by counting the losses for both types of compounds [40]. The 40 compounds detected and identified or tentatively identified are listed in Table 1, along with UV-visible and MS data. Figure 2 shows structures of several compounds identified while Figure 3, Figure 4, Figure 5 and Figure 6 show structures and full MS and MS^n^ spectra of some representative compounds. The identification of all detected and tentatively characterized compounds present in citrus fruit extracts is explained below.

Abreviattions: Glu: glucose; Neo: neohesperidose; Rut: rutinose.

#### 2.2.1. Flavonol *O*-Glycosides

Peaks **2** (Figure 3) and **3** were identified as apigenin (λ max 268, 334) derivatives. Peak **3** produced a molecular anion at *m/z* 577 and a apigenin MS^2^ fragment at *m/z* 269 (MS^3^: 225), which pointed out to the presence of apigenin 7-*O*-neohesperidoside (rhoifolin) [22]. Peak **4** with a pseudomolecular ion at *m/z* 771 (Figure 3), which in turn produced a rutin ion at *m/z* 609 (M-hexose) and MS^3^ ion at *m/z* 463 (quercetin 3-*O*-glucose, also showing a MS^4^ ion at *m/z* 301 and MS^5^ ions at *m/z* 179, 151) was proposed as an *O*-glycosylated rutin derivative (quercetin 7-*O*-glucoside-3-*O*-rutinoside). Peak **5** with a [M−H]^−^ ion at *m/z* 639 (Figure 4) and MS^2^ ions at *m/z* 315 (M-324 Daltons, di-hexose moiety) was tentatively identified as isorhamnetin-3-*O*-di-glucoside [22]. Peak **8** (Figure 5) (λ max 250, 268, 342) was identified as diosmetin 7-*O*-neohesperidoside ([M−H]^−^ ion at *m/z* 607 and MS^2^ ions at *m/z* 563: diosmetin-hexose, and *m/z* 299, diosmetin aglycone) while the isomer of the latter, peak **9** was associated to diosmetin 7-*O*-rutinoside (diosmin) (MS ions at *m/z* 463: diosmetin-hexose and 299: diosmetin) both compounds already reported in Citrus species [13,41]. Peak **12** with a [M−H]^−^ ion at *m/z* 577 (Figure 3) and MS^2^ ions at *m/z* 431 (M-rhamnose) and 269 (M-rutinose) was tentatively identified as apigenin 7-*O*-rutinoside (isorhoifolin) [22].

**Figure 7:**
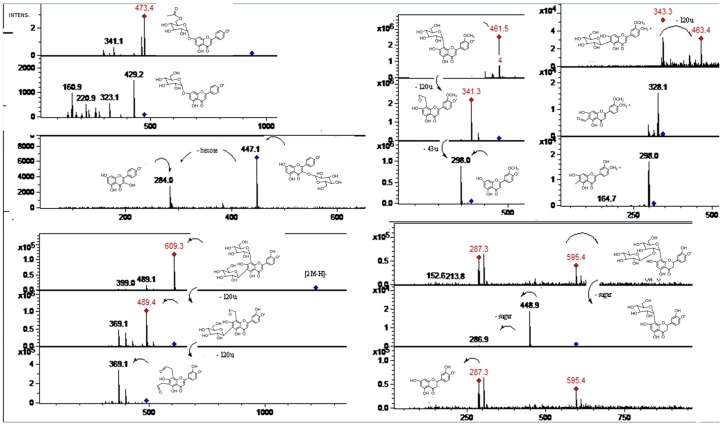
MS spectra of peaks **13**, **17**, **25**, **31**, and **33**. (Refer to Table 1).

In a similar manner, isomer compounds **19** (Figure 5) and **22** showing molecular anions at *m/z* 609 in their full MS spectra were identified as rutin (quercetin-3-*O*-rutinose, λ max 254, 354 nm), producing a daughter MS ion at *m/z* 463, by loss of rhamnose moiety and a quercetin daughter ion at *m/z* 301, with MS^3^ at *m/z* 179, 151) by loss of rutinose) [40] and hesperidin (hesperetin 7-*O*-rutinoside, λ max 285, 332 nm, hesperetin ion at *m/z* 301, by loss of rutinose) [42,43]. Peak **23** showing a [M−H]^−^ ion at *m/z* 623 (Figure 4) and a MS^2^ ion at *m/z* 315 (isorhamnetin, λ max 250, 268, 342 nm) was identified as isorhamnetin-3-*O*-rutinoside [22], while peak **24** with a molecular anion at *m/z* 477 (Figure 4) and a MS^2^ ion at *m/z* 315 was identified as isorhamnetin-3-*O*-glucoside [44,45], and peak **31** (Figure 7) ([M−H]^−^ ion at *m/z* 461, MS^2^ ions at *m/z* 563, 299 (chrysoeriol), 284) was identified as chrysoeriol 7-*O*-neohesperidoside [13]. Peak **26** (Figure 4) was identified as isorhamnetin 3-*O*-rhamnoside ([M−H]^−^ ion at *m/z* 461, MS^2^ ion at *m/z* 301, produced by loss of rhamnose moiety) [46]. Peak **29** (Figure 4) was identified as 7-*O*-methyl-isorhamnetin-3-*O*-glucoside ([M−H]^−^ ion at *m/z* 491 MS^n^ ions at *m/z* 315, 301) while peak **32** (Figure 4) was tentatively identified as quercetin-3-*O*- hexose ([M−H]^−^ at *m/z* 463, MS^2^ at *m/z* 301, produced by loss of hexose moiety) [47]. Peak **16** with two UV maxima at 262, 362 nm in the DAD spectrum and showing a molecular anion at *m/z* 593 in the negative ESI spectra (Figure 1) was identified as a kaempferol 3-*O*-rutinoside [42,48]. Cleavage of this kaempferol glycoside gave the anion aglycone at *m/z* 285 ([M−H]**^−^**) [22]. An isomer of the latter compound **16** identified with peak **14** showing a [M−H]^−^ ion at *m/z* 593 was identified as luteolin 7-*O*-hexoside (UV Max 254, 267 nm, luteolin MS ion at *m/z* 285) [41]. Peaks **21** and **30** were tentatively identified as the isomers diosmetin 7-*O*-neohesperidose and chrysoeriol 7-*O*-neohesperidose, respectively [22]. Peak **33** was identified as apigenin 7-*O*-6''acetyl-glucoside ([M−H]^−^ at *m/z* 473 and MS^2^ at *m/z* 429 (M-acetyl moiety, Figure 7) [41]. 

**Figure 8:**
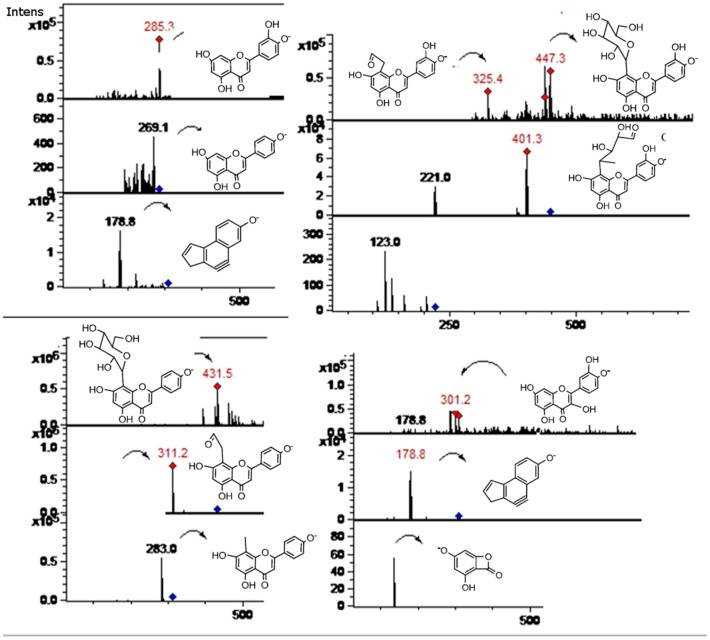
MS spectra of peaks **27**, **36**, and **37**. (Refer to Table 1).

**Figure 9:**
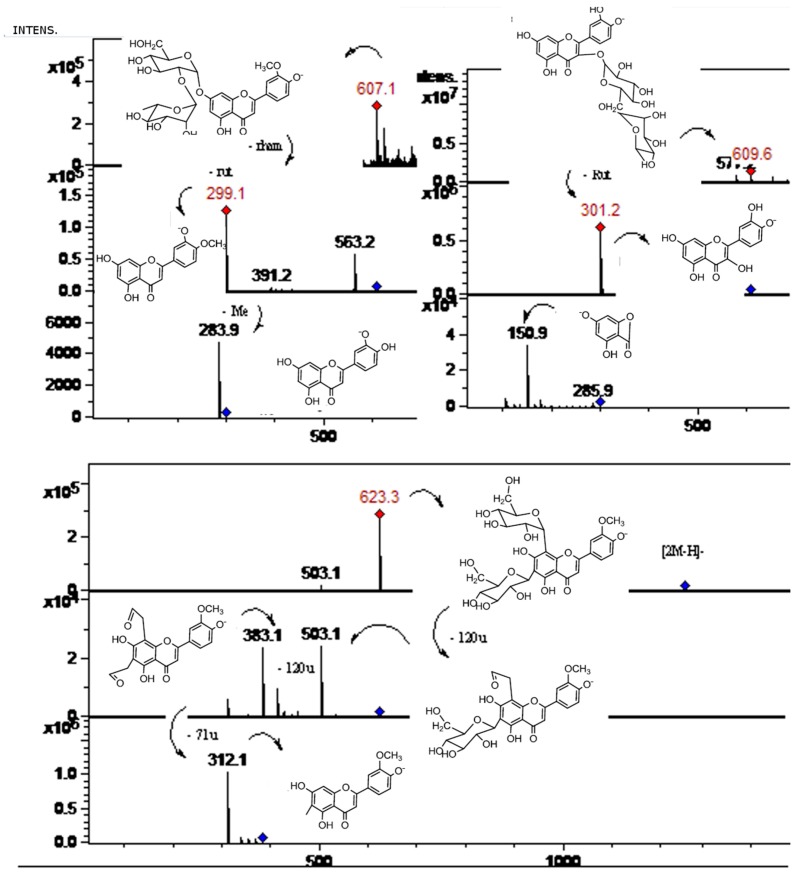
MS spectra of peaks **7**, **8**, and **19**. (Refer to Table 1).

Flavonol C-glycosides. Peaks **7**, **17** (Figure 7), and **31** were identified as C-glycosyl derivatives of diosmetin (λ max 250, 268, 342), while peaks **10** and **18** as C-glycosyl derivatives of chrysoeriol [13]. Isomer compounds identified with peaks **17** (Figure 7) and **18** both with a [M−H]^−^ ion at *m/z* 461 and characteristic MS^n^ ions at 341 (M-120) and 298 were identified as diosmetin 8-C glucoside (orientin 4'-methyl ether) and chrysoeriol 8-C-glucoside, respectively [23]. Identities of the isomers were confirmed by spiking experiments with standard compounds. In addition, related di C-glycosylated compounds identified with peaks **7** (Figure 5) and **10** showed both a pseudomolecular ion at *m/z* 623 and an adduct [2M−H]^−^ ion at *m/z* 1247. The fragmentation observed at *m/z* 503, 383, 312 (M-120, M-240) lead us to the presence of the *C*-glycosides diosmetin 6,8-di-C-glucoside and chrysoeriol 6,8-di-*C*-glucoside (stellarin-2) [40]. In a similar manner, peaks **25** (Figure 7) and **27** (Figure 8) ([M−H]^−^ ions at *m/z* 609 and 447, respectively) were tentatively identified as luteolin 6,8-di-*C*-glucoside (MS^2^ ions at *m/z* 489: M-120, 369: M-240) and luteolin 8-*C*-Glucoside [13] (MS^2^ ions at *m/z* 327: M-120 and 299), respectively. Peak **20** with a molecular anion at *m/z* 593 and typical *C*-glycosyl fragments at *m/z* 503, 473, 413 and 383 was assigned as apigenin-6,8-di-*C*-β-d-glucopyranoside (vicenin II, Figure 9), [40] identity further confirmed by spiking experiments with standard compound, while peaks **34** and **35** with pseudomolecular ions at *m/z* 431 and fragments at *m/z* 311 (M-120), 283 were identified as vitexin (apigenin-8-*C*-β-d-glucopyranoside) and isovitexin (apigenin-6-*C*-β-d-glucopyranoside), using also co-injection with standard compounds.

#### 2.2.2. Flavanone Glycoconjugates

Several known flavanones and their glycosyl derivatives were identified in Chilean Citrus (Table 1). Peak **6** was identified as eriodictyol-4'-*O*-neohesperidoside-7-*O*-glucoside [41] ([M−H]^−^ ion at *m/z* 757 and MS^2^ ions at *m/z* 595 (M-glucose), 449 (M-308, neohesperidose), and 287 (M-glucose and neohesperidose), while a related compound peak **13** (Figure 8) was identified as eriodictyol-7-*O*-neohesperidoside ([M−H]^−^ ion at *m/z* 595, MS^2^ at *m/z* 449 and 287) [13] and the isomer of the later compound peak **15** was identified as eriocitrin (eriodictyol-7-*O*-rutinose, MS^2^ at *m/z* 287: eriodictiol) [41]. Peak **28** (Figure 3) was identified as naringin (naringenin 7-*O*-neohesperidoside) ([M−H]^−^ ion at *m/z* 579, releasing a naringenin MS^2^ ion at *m/z* 271, produced by loss of a neohesperidose moiety) [23,49,50].

#### 2.2.3. Flavones C and O Glycosides

Peak **2** produced a molecular anion at *m/z* 739 and MS^n^ ions at *m/z* 431 (M-neohesperidose), and 311 (−120) and was identified asapigenin 7-*O*-neohesperidoside-6-*C*-glucoside [41].

#### 2.2.4. Flavonoid Aglycones

Peaks **36**–**39** (Figure 8) showing characteristic UV spectra and [M−H]^−^ ions at *m/z* 301, 299, 287, 269 and 285 (Table 1) were identified as the flavone aglycones quercetin, diosmetin, eriodictyol, apigenin, and luteolin respectively, [41,51,52,53]. MS-MS analysis of all of those compounds showed characteristic daughter ions (Table 1). The typical fragment ions at *m/z* 179 and 151 (Figure 3c) produced by rings A and B of the flavone structure were confirmed by RDA rearrangement of 5, 7-dihydroxy-flavones using deuterium labelling experiments [54].

#### 2.2.5. Other Compounds

Peak **1** was identified as citric acid ([M−H]^−^ ion at *m/z* 191.1)[23] while peak **11** showing a [M−H]^−^ ion at *m/z* 357 in the MS spectra (MS^2^ at *m/z* 194, 151) was identified as 3-(2-hydroxy-4- methoxyphenyl)-propanoic acid hexose, a compound previously reported in Citrus [23].

## 3. Experimental Section

### 3.1. Chemicals and Plant Material

Folin–Ciocalteu phenol reagent (2 N), Na_2_CO_3_, AlCl_3_, FeCl_3_, NaNO_2_, NaOH, quercetin, sodium acetate, HPLC-grade water, HPLC-grade acetonitrile, reagent grade MeOH and formic acid were obtained from Merck (Darmstadt, Germany). Rutin, diosmin, diosmetin, vicenin II, orientin, vitexin, isovitexin, eriodictyol, luteolin, hesperidin, naringin (all standards with purity higher than 95% by HPLC) were purchased either from ChromaDex (Santa Ana, CA, USA) or Extrasynthèse (Genay, France). Gallic acid, TPTZ (2,4,6 tri (2 pyridyl) 1,3,5 triazine), Trolox and DPPH (1,1-diphenyl-2-picrylhydrazyl radical) were purchased from Sigma-Aldrich Chemical Co. (St. Louis, MO,USA). Ripe fruits of *Citrus aurantifolia* (Christm) Swingle var. Pica (local name: Limón de Pica), (Citrus limon (L.) Burm) var Genova (local name: *Limón Genova*), *Citrus aurantifolia* (Christm) Swingle var. sutil (local name: *Limón Sutil*), were purchased at La Vega de Antofagasta fruit Market, Chile in March 2012. Examples were deposited at the Laboratorio de Productos Naturales, Universidad de Antofagasta, Antofagasta, Chile, with the numbers Cavarp-20120315 and CL-20120315 and Cavarsut-20120315, respectively.

### 3.2. Sample Preparation

Fresh citrus fruits were washed, the peels manually separated and the pulp deseeded. Each peel and pulp-juice (100 g) were separately homogenized in a blender with 100 mL of 0.1% HCl in MeOH and extracted thrice for one hour each time under dark using an ultrasonic bath. The extracts were combined, filtered and the solvent removed *in vacuo* (40 °C). The remaining aqueous partitions were separately loaded in an Amberlite XAD-7 column (5 cm × 15 cm) and rinsed with 100 mL ultrapure water. The phenolic compounds present in each partition were then eluted with 100 mL of 0.1% HCl in MeOH obtaining 0.62, 0.91 and 0.63 g of Pica, Sutil and Genova pulp extracts, respectively, and 0.76, 0.69 and 0.54 g of Pica, Sutil and Genova peel extracts, respectively. The extracts (aprox. 2 mg) were re-dissolved in 2 mL 0.1% HCl in MeOH, filtered through a 0.45 μm micropore membrane (PTFE, Waters) before use and 10 μL were injected into the HPLC instrument for analysis.

### 3.3. HPLC Analysis

A Merck-Hitachi (LaChrom, Tokyo, Japan) instrument equipped with an L-7100 pump, an L-7455 UV diode array detector, a D-7000 chromato-integrator and a column compartment was used for analyses. The sample was separated on a Purospher star-C18 column (250 mm × 5 mm, 4.6 mm i.d., Merck, Germany). The mobile phase consisted of 10% formic acid in water (A) and acetonitrile (B). A gradient program was used for HPLC-DAD and ESI-MS as follows: 90% A in the first 4 min, linear gradient to 75% A over 25 min, then linear gradient back to initial conditions for the other 15 min. The mobile phase flow rate was 1 mL/min. The column temperature was set at 25 °C; the detector was monitored at 520 nm for anthocyanins and 320–280 nm for other compounds while UV spectra from 200 to 600 nm were recorded for peak characterization.

### 3.4. Mass Spectrometric Conditions

An Esquire 4000 Ion Trap mass spectrometer (Bruker Daltoniks, Bremen, Germany) was connected to an Agilent 1100 HPLC instrument via ESI interface for HPLC-ESI-MS analysis. Full scan mass spectra were measured between *m/z* 150 and 2000 μ in positive ion mode for anthocyanins and negative ion mode for other compounds. High purity nitrogen was used as nebulizer gas at 27.5 psi, 350 °C and at a flow rate of 8 L/min. The mass spectrometric conditions for negative ion mode were: electrospray needle, 4000 V; end plate offset, −500 V; skimmer 1, −56.0 V; skimmer 2, −6.0 V; capillary exit offset, −84.6 V; and the operating conditions for positive ion mode were: electrospray needle, 4000 V; end plate offset, −500 V; skimmer 1, 56.0 V; skimmer 2, 6.0 V; capillary exit offset, 84.6 V; capillary exit, 140.6 V. Collisionally-induced dissociation (CID) spectra were obtained with a fragmentation amplitude of 1.00 V (MS/MS) using ultrahigh pure helium as the collision gas.

### 3.5. Antioxidant Assessment

#### 3.5.1. Free Radical Scavenging Activity

The free radical scavenging activity of the extracts was determined by the DPPH assay as previously described [55], with some modifications. DPPH radicals absorb at 517 nm, but upon reduction by an antioxidant compound, absorption decreases. Briefly, 50 μL of processed SPE MeOH extract or pure compound prepared at different concentrations was added to 2 mL of fresh 0.1 mM solution of DPPH in methanol and allowed to react at 37 °C in the dark. After thirty minutes the absorbance was measured at 517 nm. The DPPH scavenging ability as percentage was calculated as: DPPH scavenging ability = (A_control_ − A_sample_/A_control_) × 100. Afterwards, a curve of % DPPH bleaching activity *versus* concentration was plotted and IC_50_ values were calculated. IC_50_ denotes the concentration of sample required to scavenge 50% of DPPH free radicals. The lower the IC_50_ value the more powerful the antioxidant capacity. If IC_50_ ≤ 50 μg/mL the sample has high antioxidant capacity, if 50 μg/mL < IC_50_ ≤ 100 μg/mL the sample has moderate antioxidant capacity and if IC_50_ > 200 μg/mL the sample has no relevant antioxidant capacity. Gallic acid (from 1.0 to 125.0 μg/mL, R^2^ = 0.991) and quercetin (from 1.0 to 125.0 μg/mL, R^2^ = 0.993) were used as standard antioxidant compounds, and were determined to have IC_50_ values of 1.1 µg/mL (6.8 µmol/L) and 7.5 µg/mL (24.8 µmol/L), respectively.

#### 3.5.2. Ferric Reducing Antioxidant Power

The determination of ferric reducing antioxidant power or ferric reducing ability (FRAP assay) of the extracts was performed as described by [56] with some modifications. The stock solutions prepared were 300 mM acetate buffer pH 3.6, 10 mM TPTZ (2,4,6-tri (2-pyridyl)-s-triazine) solution in 40 mM HCl, and 20 mM FeCl_3_·6H_2_O solution. Plant extracts or standard methanolic Trolox solutions (150 μL) were incubated at 37 °C with 2 mL of the FRAP solution (prepared by mixing 25 mL acetate buffer, 5 mL TPTZ solution, and 10 mL FeCl_3_·6H_2_O solution) for 30 min in the dark. Absorbance of the blue ferrous tripyridyltriazine complex formed was then read at 593 nm. Quantification was performed using a standard calibration curve of antioxidant Trolox (from 0.2 to 2.5 μmol/mL, R^2^: 0.995). Samples were analyzed in triplicate and results are expressed in μmol TE/gram dry mass.

#### 3.5.3. Superoxide Anion Scavenging Activity

The enzyme xanthine oxidase is able to generate superoxide anion radical (O_2_^.−^) “*in vivo*” by oxidation of reduced products from intracellular ATP metabolism. The superoxide anion generated in this reaction sequence reduces the nitro blue tetrazolium dye (NBT), leading to a chromophore with a maximum of absorption at 560 nm. Superoxide anion scavengers reduce the speed of generation of the chromophore. The Superoxide anion scavenging activities of isolated compounds and fractions were measured spectrophotometrically in a microplate reader as reported previously [47]. All compounds, and berry extracts were evaluated at 100 μg/mL. Values are presented as mean ± standard of three determinations.

### 3.6. Polyphenol, Flavonoid and Vitamin C Contents

The total polyphenolic contents (TPC) of *citrus* fruits were determined by the Folin-Ciocalteau method [34] with some modifications. An aliquot of each processed SPE extract (200 μL), was added to the Folin–Ciocalteau reagent (2 mL, 1:10 v/v in purified water) and after 5 min of reaction at room temperature (25 °C), 2 mL of a 100 g/L solution of Na_2_CO_3_ was added. Sixty minutes later the absorbance was measured at 710 nm. A calibration curve was performed with the standard gallic acid (concentrations ranging from 16 to 500 μg/mL, R^2^ = 0.999) and the results expressed as mg gallic acid equivalents/g dry mass.

Determination of total flavonoid content (TFC) of the methanolic extracts was performed as reported previously [57] using the AlCl_3_ colorimetric method. Quantification was expressed by reporting the absorbance in the calibration graph of quercetin, which was used as the flavonoid standard (from 0.1 to 65.0 μg/mL, R^2^ = 0.994). Results are expressed as mg quercetin equivalents/g dry mass. Vitamin C content was determined by redox titration with a potassium iodate solution, after the samples were homogenized and acidized with sulphuric acid (20%) and in the presence of IK, using starch as an indicator. The end point of the reaction was the appearance of a blue colour [31]. The ascorbic acid content was expressed as mg of ascorbic acid per 100 g fresh weight (FW). All spectrometric measurements were performed using a Unico LQ2800 UV-vis spectrophotometer (Unico instruments, Co., Ltd., Shanghai, China) or a Pharo spectroquant 300 (Merck, Darmstadt, Germany).

### 3.7. Statistical Analysis

The statistical analysis was carried out using the originPro 9.0 software packages (Originlab Corporation, Northampton, MA, USA). The determination was repeated at least three times for each sample solution. Analysis of variance was performed using ANOVA. Significant differences between means were determined by Student’s *t*-test (*p* values <0.05 were regarded as significant).

## 4. Conclusions

The LC-UV and ESI-MS^n^ system used in this work allowed the detection and the tentative identification of 40 phenolic compounds in three edible pulps and peels of citrus fruits widely consumed in Chile. Several flavonoids including one kaempferol-*O*-hexoside (peak **16**) and one hesperidin derivative (peak **22**) three quercetin derivatives (peaks **4**, **19** and **36**), five isorhamnetin derivatives (peaks **5**, **23**, **24**, **26** and **29**) four luteolin derivatives (peaks **14**, **25**, **27** and **40**), seven apigenin derivatives (peaks **2**, **3**, **12**, **20**, **34**, **35** and **39**), seven diosmetin derivatives (peaks **7**–**9**, **17**, **21**, **31** and **37**), three chrysoeriol derivatives (peaks **10**,**18** and **30**), and four eryodictiol derivatives (peaks **6**, **1315** and **38**) were identified in negative and positive mode using full scan mass measurements and MS^n^ fragmentations. The results obtained pointed out that the methodology developed is appropriate for rapid analysis and identification of C and O-glycosil flavonoids in extracts from native citrus fruits and can be potentially used for other edible South American fruits. The highest total phenolic content was found in Pica peels which also showed the highest total flavonoid content (34.59 ± 0.81 mg/g GAE dry weight and 23.06 ± 1.57 mg quercetin/g dry weight, respectively). The vitamin C pattern for citrus species was similar but with higher content in *Genova* pulps, followed by the pulp of the variety *Sutil* (60.13 ± 1.28 and 56.53 ± 1.06 mg ascorbic acid g dry weight, respectively). The antioxidant capacity was also higher for Pica peels (10.34 ± 1.23 µg/mL in the DPPH assay and 120.63 ± 2.45 µM trolox equivalents/g dry weight in the FRAP assay).The antioxidant properties and high content of flavonoids in the peels can make this waste material a good source of nutraceutical and healthy phenolic compounds, especially to be used as anti-ageing products, due to the high content of polyphenols. However, further research is needed to explore other biological activities of *Citrus* peels to support their potential in the human diet.

## Supplementary Materials

Supplementary materials can be accessed at: http://www.mdpi.com/1420-3049/19/11/17400/s1.

## Supplementary Files

Supplementary File 1
