# Supplementary material for: HPLC-UV-MS Profiles of Phenolic Compounds and Antioxidant Activity of Fruits from Three Citrus Species Consumed in Northern Chile
Source: Molecules. 2014 Oct 29;19(11):17400–21. doi: 10.3390/molecules191117400 (PMC6271594; doi:10.3390/molecules191117400)

Supplementary Materials

Table S1. Total Phenolic Content (TPC), Total Flavonoid Content (TFC), Ascorbic acid Content (AA), Scavenging of the 1,1-diphenyl-2-picrylhydrazyl Radical (DPPH), Ferric Reducing Antioxidant Power (FRAP), Superoxide Anion scavenging activity (SA), and Extraction Yields of Three Edible Citrus Fruits from the II Region of Chile.

Species and Plant Part	TPC (mg/g) $^{\alpha}$	TFC (mg/g) $^{\beta}$	AA (mg/g) $^{\gamma}$	DPPH (IC_{50}, μg/mL) $^{\delta}$	FRAP(μmol/g) $^{\varepsilon}$	SA (mg/g) ^{o}	w/w Extraction Yield(%) $^{\mu}$
Pica peel (ppe)	34.59 ± 0.81	23.06 ± 1.57	36.19 ± 0.38	10.34 ± 1.23p	120.63 ± 2.45	76.63 ± 2.14x	0.76
Pica pulp (ppu)	18.58 ± 0.62	11.71 ± 1.62abe	49.49 ± 0.32	12.98 ± 1.45p	112.31 ± 1.13	65.05 ± 1.49pqr	0.62
Genova peel (gepe)	22.69 ± 1.23	13.44 ± 1.22c	34.21 ± 1.77	20.11 ± 1.32q	86.5 ± 1.07z	63.3 ± 2.0rstw	0.63
Genova pulp (gepu)	9.47 ± 0.92	8.37 ± 0.89adf	60.13 ± 1.28	27.37 ± 1.42u	74.78 ± 0.48	59.03 ± 1.96vw	0.54
Sutil peel (supe)	25.58 ± 1.02	15.5 ± 1.03bcde	42.38 ± 1.21	16.59 ± 1.18r	98.79 ± 1.45	67.30 ± 1.38ps	0.91
Sutil pulp (supu)	14.66 ± 1.10	11.13 ± 0.96f	56.53 ± 1.06	18.73 ± 1.22qr	92.02 ± 1.14	60.35 ± 0.91qtv	0.69
GA $^{\Phi}$	-	-	-	6.61 ± 0.23	148.12 ± 2.21	94.39 ± 1.98	-
Rutin $^{\Phi}$	-	-	-	24.05 ± 0.85u	84.6 ± 1.20z	76.85 ± 1.71x	-

$^{\alpha}$ Total phenolic content (TPC) expressed as mg gallic acid/g dry weight. $^{\beta}$ Total flavonoid content (TFC) expressed as mg quercetin/g dry weight. $^{\gamma}$ Ascorbic acid content expressed as mg vitamin C/g dry weight. $^{\delta}$ Antiradical DPPH activities are expressed as IC_{50} in μg/mL for extracts and compounds. $^{\Phi}$ Used as standard antioxidant. $^{\varepsilon}$ Expressed as μM trolox equivalents/g dry weight. ^{o} Expressed in percentage scavenging of superoxide anion at 100 μg/mL. $^{\mu}$ Extraction yields expressed in percent W/W extraction after SPE, on the basis of fresh material. Values in the same column marked with the same letter are not significantly different (at $p < 0.05$).

(Abreviations: ppe: pica peel, ppu: pica pulp, supe: sutil peel, supu: sutil pulp, gepe: genova peel; gepu: genova pulp, GA: gallic acid).

Figure S1. Correlation curves between TPC and DPPH.

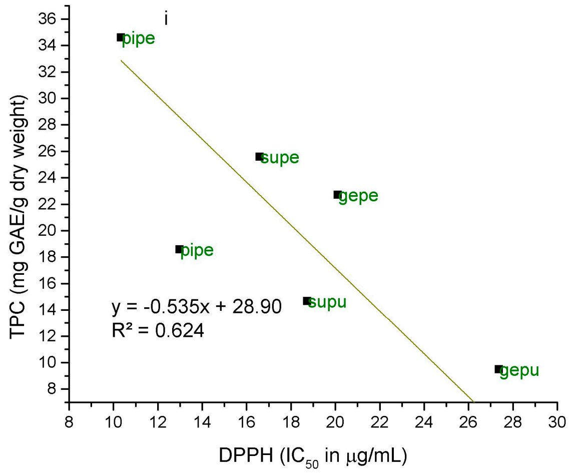

Figure S2. Correlation curves between TFC and DPPH.

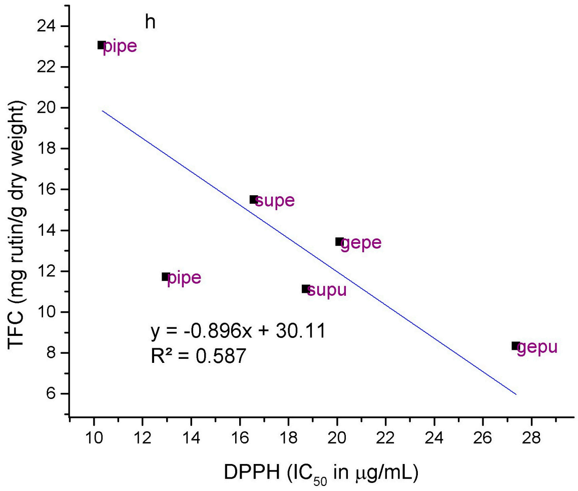

Figure S3. Correlation curves between DPPH and SA.

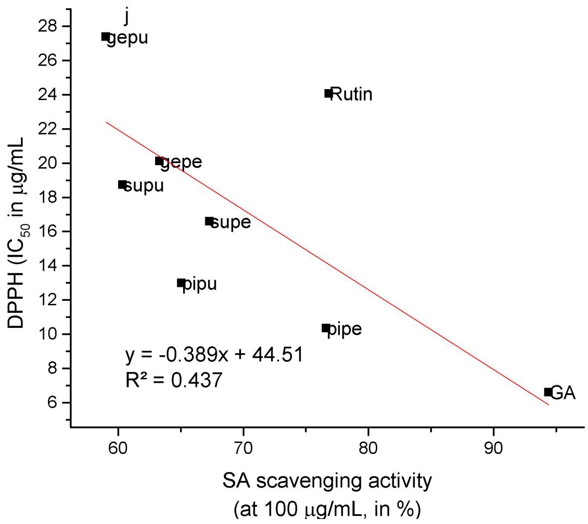

Figure S4. Correlation curves between FRAP and SA.

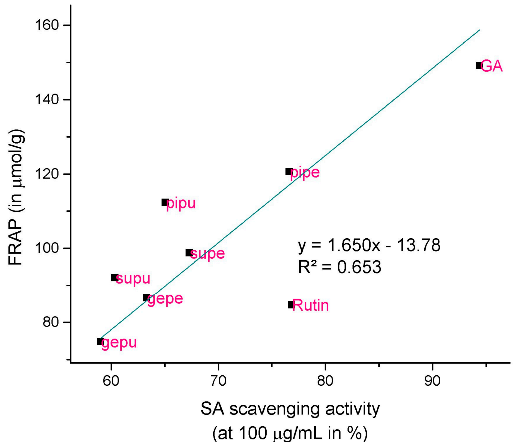

Figure S5. Correlation curves between DPPH and FRAP.

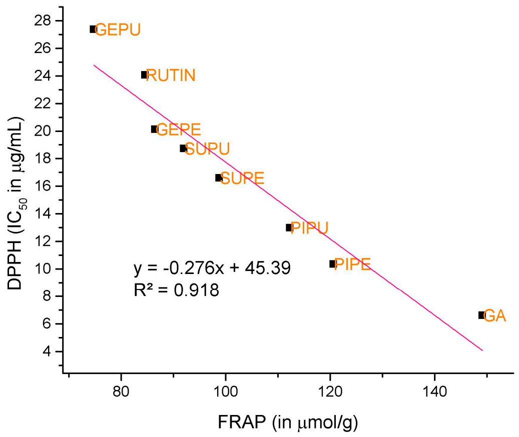

Figure S6. Zoom of the chromatograms of Pica lemon extracts. Pica peel (upper chromatogram) and Pica pulp (lower chromatogram). Major compounds detected in this species are: Eriodictyol-4'-*O*-neohesperidoside-7-*O*-glucoside (compound **6**), Eriodictyol-7-*O*-neohesperidoside (**13**), Luteolin 7-*O*-rutinoside (**14**), Hesperidin (**22**), Isorhamnetin-3-*O*-glucoside (**24**), orientin (**27**), Apigenin (**39**), luteolin (**40**).

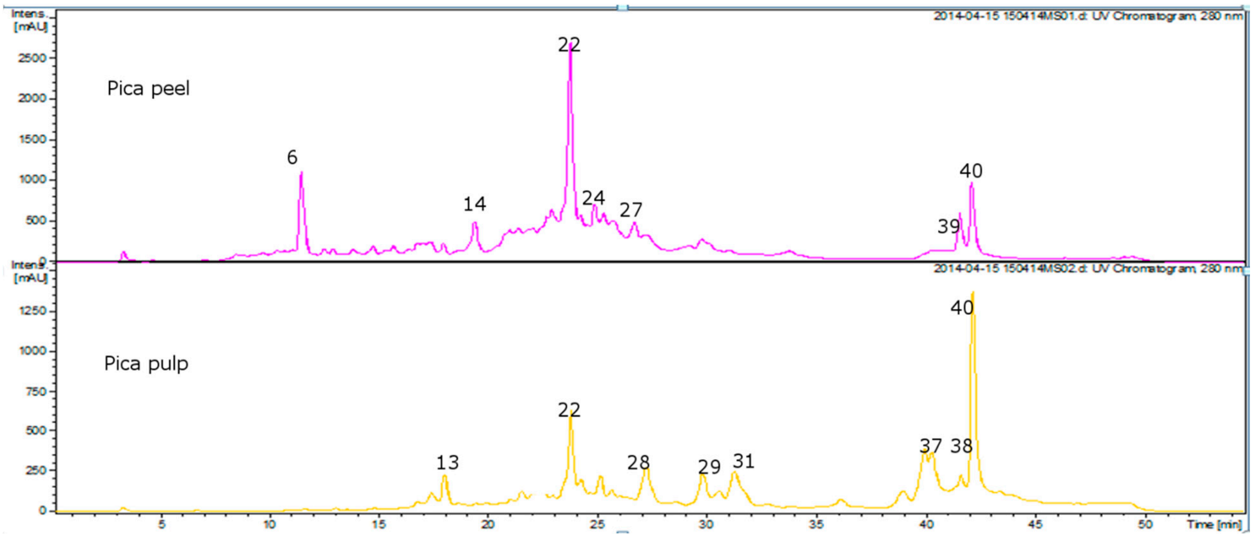

Figure S7. Zoom of the chromatograms of Genova lemon extracts. Genova peel (upper chromatogram) and Genova pulp (lower chromatogram). Major compounds detected in this species are: Diosmin (compound **16**), Vicenin-2 (**20**), Hesperidin (**22**), Apigenin (**39**), luteolin (**40**).

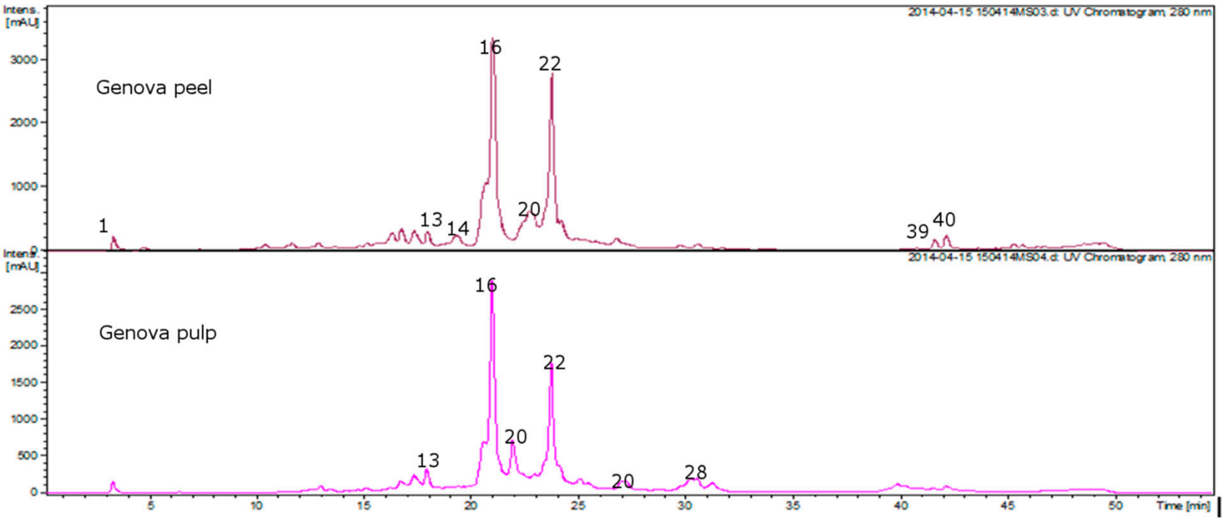

Figure S8. Zoom of the chromatograms of Sutil lemon extracts. Sutil peel (upper chromatogram) and Sutil pulp (lower chromatogram). Major compounds detected in this species are: Eriodictyol-4'-*O*-neohesperidoside-7-*O*-glucoside (compound **6**), 3-(2-hydroxy-4-methoxyphenyl)-propanoic acid hexose (**11**), diosmin (compound **16**), hesperidin (**22**), diosmetin (**37**), eriodictyol (**38**), apigenin (**39**), luteolin (**40**).

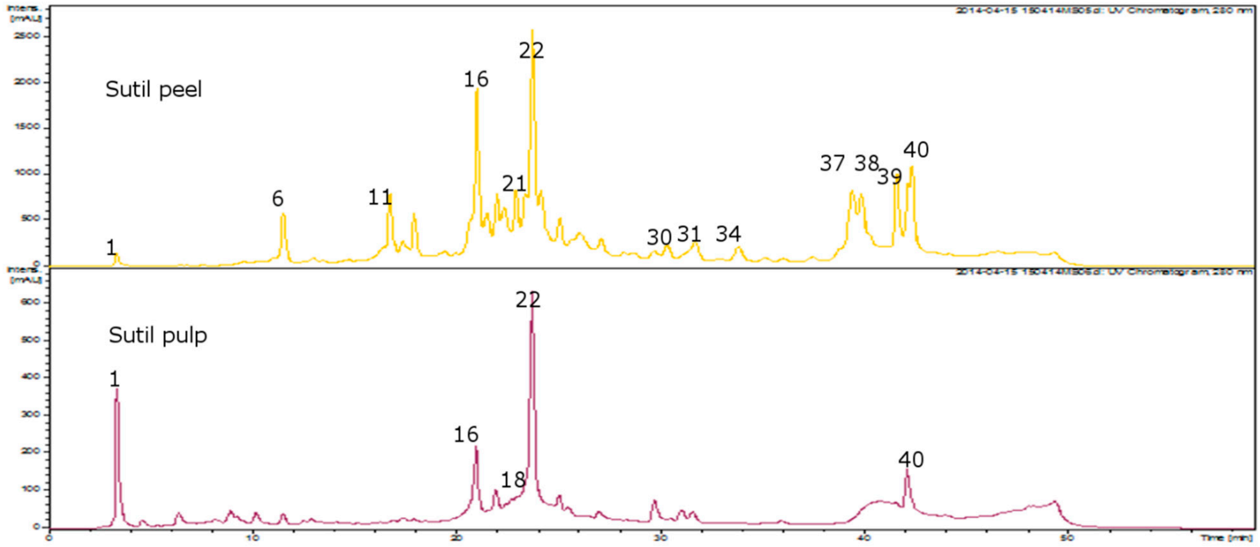

Supplement: Supplementary File 1 [file molecules-19-17400-s001.pdf]
